# Unravelling paralogous gene expression dynamics during three-spined stickleback embryogenesis

**DOI:** 10.1038/s41598-019-40127-2

**Published:** 2019-03-06

**Authors:** Elisavet Kaitetzidou, Ioanna Katsiadaki, Jacques Lagnel, Efthimia Antonopoulou, Elena Sarropoulou

**Affiliations:** 1 Department of Zoology, School of Biology, Faculty of Sciences, Aristotle University of Thessaloniki, Thessaloniki, Greece; 2 Institute for Marine Biology, Biotechnology and Aquaculture (IMBBC), Hellenic Centre for Marine Research (HCMR), Heraklion, Greece; 3 Centre for Environment Fisheries and Aquaculture Science, (Cefas), Weymouth, UK; 4 Institut National de la Recherche Agronomique (INRA), Génétique et Amélioration des Fruits et Légumes (GALF), Montfavet Cedex, France

## Abstract

Development requires the implementation of a plethora of molecular mechanisms, involving a large set of genes to ensure proper cell differentiation, morphogenesis of tissues and organs as well as the growth of the organism. Genome duplication and resulting paralogs are considered to provide the raw genetic materials important for new adaptation opportunities and boosting evolutionary innovation. The present study investigated paralogous genes, involved in three-spined stickleback (*Gasterosteus aculeatus*) development. Therefore, the transcriptomes of five early stages comprising developmental leaps were explored. Obtained expression profiles reflected the embryo’s needs at different stages. Early stages, such as the morula stage comprised transcripts mainly involved in energy requirements while later stages were mostly associated with GO terms relevant to organ development and morphogenesis. The generated transcriptome profiles were further explored for differential expression of known and new paralogous genes. Special attention was given to *hox* genes, with *hoxa13a* being of particular interest and to pigmentation genes where *itgb1*, involved in the melanophore development, displayed a complementary expression pattern throughout studied stages. Knowledge obtained by untangling specific paralogous gene functions during development might not only significantly contribute to the understanding of teleost ontogenesis but might also shed light on paralogous gene evolution.

## Introduction

The three-spined stickleback (*Gasterosteus aculeatus*), a small fresh-water teleost species, has been used for many years as a fish model species. Studies regarding three-spined stickleback developmental stages were significantly enhanced by the ability to manipulate spawning in captivity^1^. To date and to the best of our knowledge, gene expression analysis during ontogenesis was performed only in the late developmental stages of the three-spined stickleback (3 days post-hatching, dph) after maternal exposure to predation risk^2^. Investigations of the molecular background during the early development of the three-spined stickleback involved only the expression of specific genes, such as *hox* genes, in relation to the axial formation of the embryos^3^, as well as of *fgf8* co-orthologs^4^. In non-mammalian vertebrates, a considerable number of developmental studies were performed using the model fish, zebrafish (*Danio rerio*)^5,6^. Nevertheless, zebrafish has been mainly used as a model species in human medical research, and may not be sufficient to unravel all differences in the developmental processes among vertebrates and especially among teleosts^7,8^. In addition, the phylogenetic position of the three-spined stickleback^9^ may be of advantage for knowledge transfer to other species belonging to the Eupercaria which comprise most of the prevalent species in Mediterranean aquaculture.

With regard to the molecular toolbox of the three-spined stickleback, an excess of genome, as well as transcriptome data, has been continuously accumulated. Yet, mis- or un-annotated genes may be present, especially considering the teleost-specific whole genome duplication (TGD) event. The TGD has been estimated to have occurred 320–400 Ma ago^10,11^ and about 15% of the duplicated genes, namely paralogs, have been retained in the genome of teleosts^12^. In addition, duplicated genes may undergo functional divergence and altered selective constraints^13,14^. Consequently, preserved paralogs may either maintain the same function, a quota of the original function (sub-functionalization), or acquire a completely new role (neo-functionalization)^15^. These processes underline the importance of investigating paralogous genes in a broad range of biological processes, and especially teleost-specific paralogs are discussed in functional studies which investigated their expression in, for instance, sex determination^16^, hormonal system^17^, osmoregulation^18^ as well as during development^15,19^.

In the present study, we examined the transcriptomic profiles of five key developmental stages in the three-spined stickleback, ranging from early morula to 24 hours post-hatching (hph). Therefore, high throughput sequencing was carried out followed by transcript annotation and differential gene expression analysis. The subsequent enrichment analysis aimed tο reveal the presence of known and unknown genes playing a pivotal role during development. The generated transcriptome profiles were further explored for known and unknown paralogous genes differentially expressed during three-spined stickleback development. Emphasis was given to two groups of genes: *hox* genes, a group of duplicated genes with a fundamental role in the development of all bilateral animals, as well as to a group showing high expression plasticity, i.e., genes involved in body pigmentation processes.

## Results

### Data description

Sequencing of 15 libraries corresponding to three biological replicates of the five developmental stages, produced ~275 million reads. After quality trimming, ~230 million reads (~85%) were used for downstream analysis (Supplementary Table S1). The majority of the trimmed reads were successfully mapped to the genome of the three-spined stickleback with an average percentage among stages of ~77%. Successfully mapped reads were used to generate a transcriptome assembly consisting of 101,792 transcripts. The generated transcriptome assembly (Supplementary Table S2) was used as a reference transcriptome in the present study. Out of the 101,792 mapped-to-genome transcripts, 22,892 (~22.5%) were assigned to three-spined stickleback genes already identified and characterized. Blastx search against the nr database of NCBI of the generated reference transcriptome resulted in 37,413 (~36.7%) annotated transcripts.

### Evaluation of obtained data matrix (read counts)

**Figure 1:**
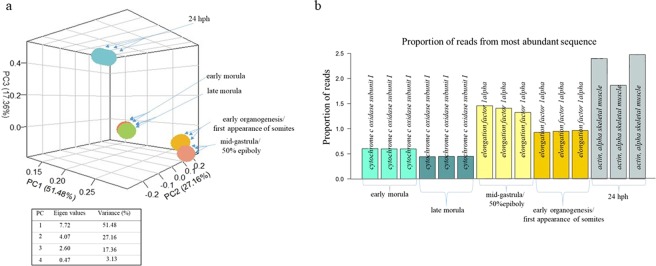
(**a**) Principal component analysis of developmental stages and (**b**) the most abundant sequence of each sample, as a proportion of the total reads. (**a**) PCA grouped together the 3 biological replicates for each developmental stage. Eigen values, as well as the percentage of variance covered by the first four components, are also shown. Three principal components with cumulative percentage of variance explained: >95%, were chosen. (**b**) Replicates of each stage, as well as neighbouring stages, displayed identical most abundant transcripts. These transcripts were: *cytochrome oxidase subunit I* for both early and late morula stage, for mid-gastrula/50% epiboly and early organogenesis/first appearance of somites and actin alpha, skeletal muscle for the last studied stage: 24 hph.

Evaluation of the read counts was performed by two different approaches: (i) PCA analysis (Fig. 1a) and (ii) bar plots illustrating arithmetic information relevant to transcripts (most abundant transcripts, null counts, and sum of all counts) (Figs 1b and S1). PCA analysis revealed that the three replicates of early morula and the three replicates of late morula had almost identical principal component coordinates (Fig. 1a). On the contrary, replicates of the other three stages were well separated.

Concerning transcript abundance analysis, *cytochrome oxidase subunit I* was identified as the most abundant transcript in early and late morula. Similarly, *elongation factor 1-alpha* (efα) was found to be most abundant in mid-gastrula/50% epiboly and early organogenesis/first appearance of somites, while in the 24 hph stage, *actin-alpha skeletal muscle* was found to be the most abundant transcript (Fig. 1b). Additionally, the proportion of null counts, as well as total read counts, were similar among the replicates of each stage (Supplementary Fig. S1).

### Differential expression analysis

**Figure 2:**
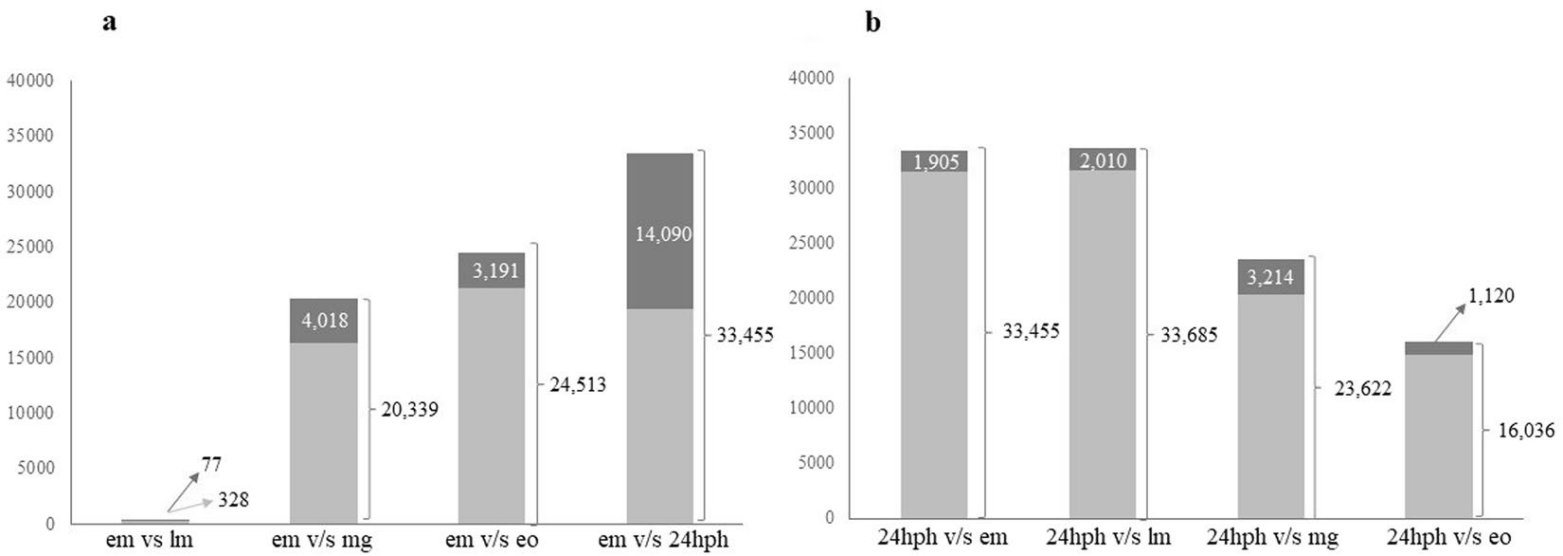
Number of differentially expressed transcripts between two extreme and the rest of the four stages studied. (**a**) Differentially expressed transcripts between the first studied stage (early morula) or (**b**) the last studied stage (24 hph) and the remaining four stages. The number of unique transcripts differentially expressed are shown in dark grey.

Pairwise comparison of each stage with the other four stages (loop-design) resulted in 10 datasets (one for each comparison). The number of transcripts after each comparison for different padj and log2 fold change ≥|2| is shown in Table 1. In the present study the most stringent parameters (transcripts with padj <0.005 and with log2 fold change ≥|2|) were considered as differentially expressed. Hierarchical clustering of all differentially expressed transcripts grouped early developmental stages under one sub-branch, the next two under a second sub-branch and the last studied stage (24 hph) was placed on its own in a separate branch (Supplementary Fig. S2). Differentially expressed transcript abundance between the four stages and the two extreme stages studied is shown in Fig. 2 i.e., between the four stages and the early morula (Fig. 2a) and between the four stages and 24 hph (Fig. 2b). Early and late morula comparison produced the lowest number of differentially expressed transcripts (328) while comparing early morula with 24 hph resulted in the highest number of differentially expressed transcripts (33,455). The number of differentially expressed transcripts between 24 hph and early and late morula were similar, while numbers between 24 hph and early organogenesis/first appearance of somites were the lowest among all the comparisons made with 24 hph as a reference (Fig. 2b). Having early morula instead of 24 hph as the reference stage produced higher numbers of unique differentially expressed transcripts (presented in dark grey in Fig. 2a) characterizing each pairwise comparison.

**Table 1:** Number of transcripts differentially expressed between stages for different padj threshold as well as for log2 fold change ≥|2|.

	late morula				mid-gastrula/50% epiboly				early organogenesis/first appearance of somites				24 hph			
padj log2 fold change	<0.05	<0.01	<0.005	<0.005 ≥|2|	<0.05	<0.01	<0.005	<0.005 ≥|2|	<0.05	<0.01	<0.005	<0.005 ≥|2|	<0.05	<0.01	<0.005	<0.005 ≥|2|
early morula	6,662	4,607	4,114	328	35,447	30,538	29,015	20,339	42,820	37,133	33,876	24,513	49,028	43,315	39,925	33,455
late morula					35,255	30,414	28,867	20,390	41,136	35,661	33,909	24,744	47,602	41,835	39,984	33,685
mid-gastrula/50% epiboly									24,220	19,565	18,234	8,087	38,611	32,709	30,850	23,622
early organogenesis/first appearance of somites													32,227	26,420	24,685	16,036

**Figure 3:**
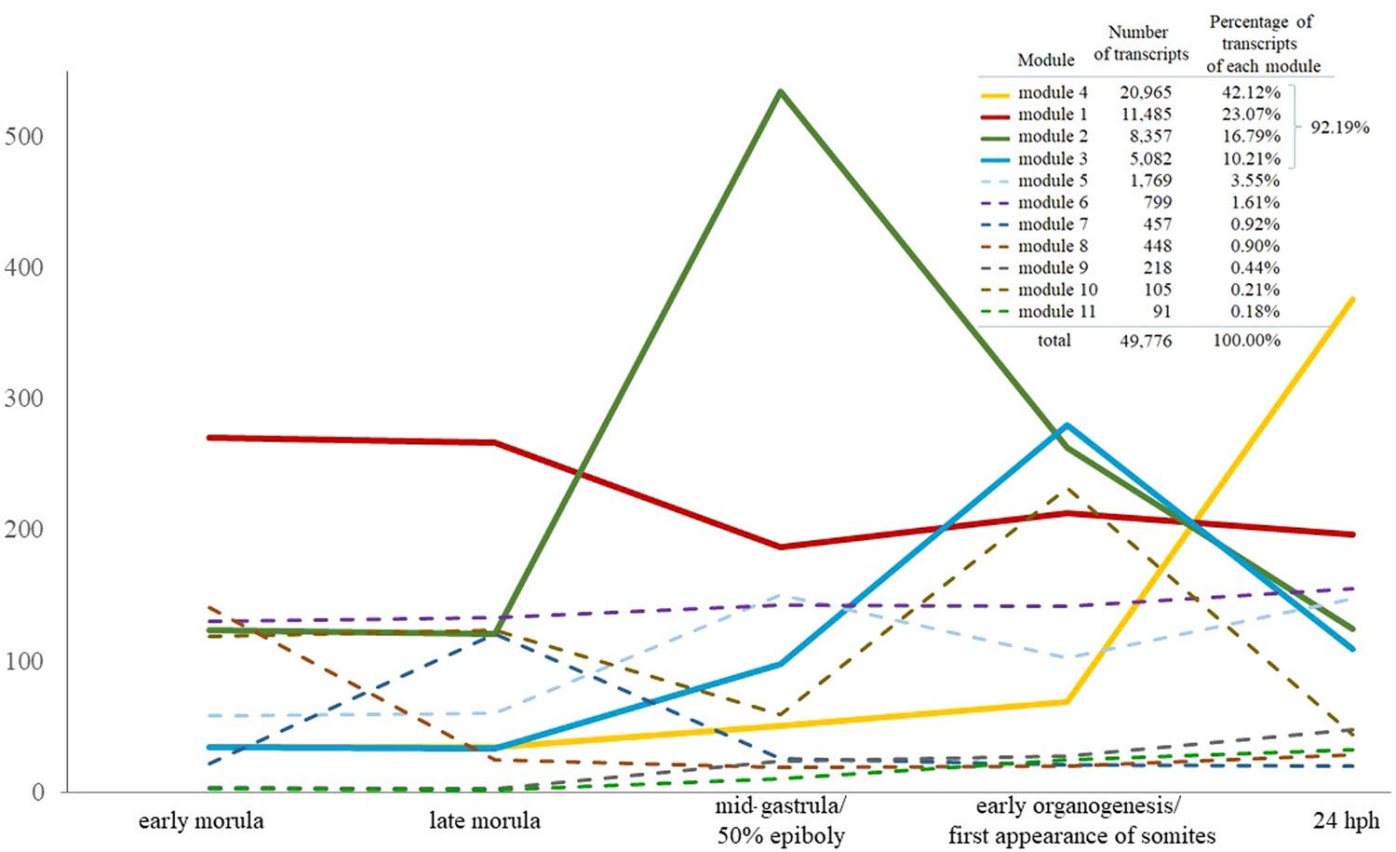
The expression pattern of differentially expressed transcripts of each of the 11 modules. Module 1, 2, 3, and 4 are shown in bold lines and were further analysed with enrichment analysis. Expression pattern corresponding to the rest of the modules are shown as discontinuous lines. Y-axis shows the average normalized counts number of all transcripts of each module. The total number of transcripts included in each module as well as their percentage against all clustered transcripts are also shown.

**Figure 4:**
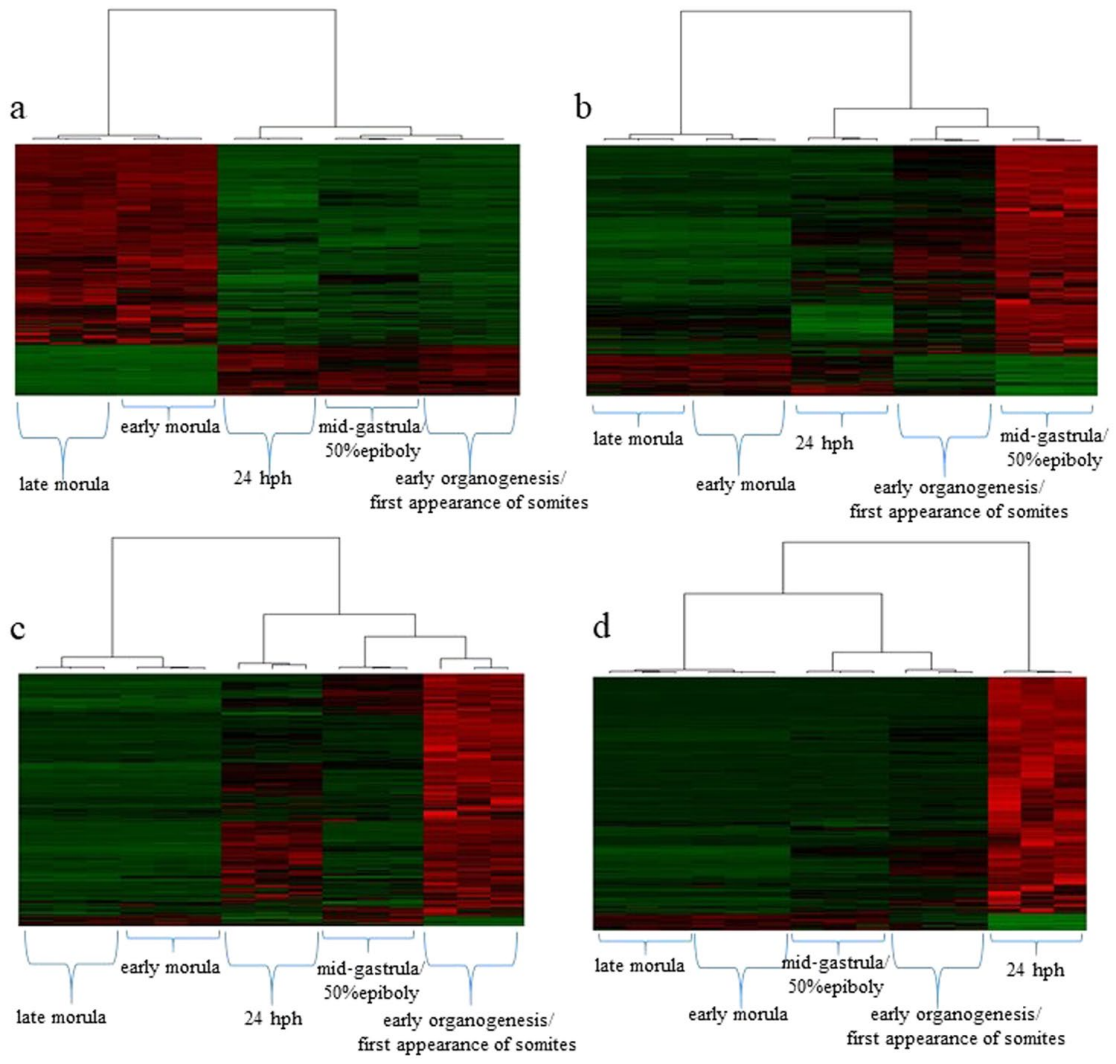
Heat maps illustrating the expression pattern of differentially expressed transcripts of modules (**a**) 1, (**b**) 2, (**c**) 3 and (**d**) 4. Each row corresponds to a transcript and each column to a biological sample. Upregulation is shown in red colour while downregulation is shown in green.

Differentially expressed transcripts were grouped into 11 modules comprising 49,776 transcripts (99.76% of differentially expressed transcripts). About 92% of all transcripts were represented in the first four modules (Fig. 3). Moreover, the average of the expression of transcripts from each module through the developmental stages is shown. Bold lines correspond to four different modules consisting of transcripts that were mainly upregulated either in early and late morula stages (module 1), or mid-gastrula/50% epiboly stage (module 2), or early organogenesis/first appearance of somites stage (module 3) or 24 hph stage (module 4). The expression patterns of each transcript of the four modules are illustrated in the form of heat maps in Fig. 4.

### Meta-analysis of differentially expressed transcripts

**Figure 5:**
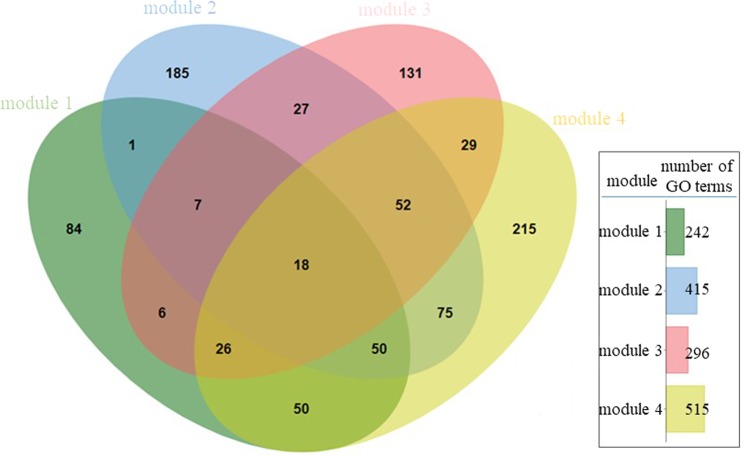
Venn diagram of GO terms under biological process, which are included in modules 1, 2, 3, and 4. The total number of GO terms included in each of the above modules is shown.

Modules 1, 2, 3 and 4 were further studied by enrichment analysis. Therefore each of the modules served as a test set and all of the annotated transcripts as a reference set. Enrichment analysis resulted in distinct GO terms, for each of the modules (Supplementary Table S3). Shared and unique GO terms which are present in the four modules are visualized in the form of a Venn diagram (Fig. 5). Module 4 (transcripts mainly upregulated at 24 hph) included the highest number of GOs, followed by module 2 (transcripts upregulated at mid-gastrula/50% epiboly), module 3 (transcripts upregulated at early organogenesis/first appearance of somites) and module 1 (transcripts upregulated at early and late morula). Unique GO terms found in each of the modules 1, 2, 3 and 4 are listed in Supplementary Table S4.

### Paralog identification in the transcriptome of three-spined stickleback embryos

In total 2,455 candidate paralogs were identified among differentially expressed transcripts (Supplementary Table S5). Paralogous genes, known to have a decisive role in embryogenesis and later development from studies in other teleost species, were searched in our transcriptome and are shown in Tables 2, 3 and Supplementary Table S6. Table 2 contains transcripts with a role in development for which both paralogs were found. On the other hand, Table 3 contains transcripts that were found only in a single copy in the generated transcriptome of the present study but do have a paralogous pair, if not in the stickleback, then in another fish species. Transcripts expressed differentially between at least two stages are noted with an asterisk.

**Table 2:** Transcripts annotated as paralogous genes with a known role in teleost development, where both paralogs were detected in the present study.

Gene ID	Chr	References	Gene ID	Chr	References
*barhl1* (−/*)	scf. 1455	^51^	*bmp1* (*/*)	XIII	^52^
	XIV			XIV	
*bmp7* (*/*)	XVII	^53^	*casq1* (*/*)	III scf. 713	^54^
	scf. 68				
*casq2* (−/*)	XVI	^54^	*dmbx1* (*/*)	III	^55^
	I			VIII	
*ext1* (*/*)	X	^56^	*foxc1* (*/*)	III	^57^
	XX			VIII	
*gpi* (*/*)	II	^58^	*foxb1* (*/*)	XIX	^57^
	XIX			II	
*heyI* (*/*)	XX	^59^	*hce1* (*/*/*)	XVIII	^60^
				scf. 48	
	X			scf. 692	
*jamb* (−/*)	I	^61^	*jamc* (*/*)	VII	^61^
	XVI			I	
*nanos1* (*/*)	IV	^62^	*nrf1* (*/−)	V	^63^
	XV			XI	
*pax2* (−/*)	VI	^64^	*ppar alpha* (*/*)	IV	^65^
	V			XIX	
*tle3 (groucho3)* (*/−)	XIX	^66^	*snai1* (*/−)	XVII	^67^
	II			scf. 180	
*wnt4* (*/*)	XVII	^68^	*pou2f2* (*/−)	XX	^69^
	XX			X	
*pou3f2* (*/*)	XX	^69^	*stat1* (*/*)	I	^70^
	XVIII			XVI	
*tbx5* (*/*)	XIV	^71^	*lbx1* (*/*)	IX	^72^
	XIII			VI	
*wt1* (*/*)	XIX	^73^	*adam17* (*/−)	scf. 269	^21^
	II			XV	
*hdac1* (*/*)	X	^21^	*en1* (*/*)	XVI	^21^
	XV			I	
*gja5* (*/−)	VI	^21^	sox9 (*/*)	V	^21^
	XVI			XI	
*tyr* (*/*)	I	^21^	*mitf* (*/−)	XVII	^21^
	VII			XII	
*rab38* (*/*)	I	^21^	*erbb3* (*/*)	scf. 27	^21^
	VII			XII	
*silv (pmel)* (*/*)	scf. 27	^21^	*itgb1* (*/*)	XXI	^21^
	XII			scf. 169	
*mcoln3* (−/*)	VIII	^21^	*spr* (*/*)	XIII	^21^
	III			XIV	
*csf1r* (*/*)	IV	^21^	*ghr* (*/*)	XIII	^21^
	VII			XIV	
hoxa2a*	X	^31^	hoxa9a*	X	^31^
hoxa2b*	XX		hoxa9b*	XX	
hoxa10a*	X	^31^	hoxa11a*	X	^31^
hoxa10b*	XX		hoxa11b*	XX	
hoxa13a*	X	^31^	hoxb5a*	XI	^31^
hoxa13b*	XX		hoxb5b*	V	
hoxd4a*	XI	^31^	hoxd9a*	XI	^31^
hoxd4b*	VI		hoxd9b*	VI	

Transcripts that were differentially expressed between at least two stages are noted with an asterisk.

**Table 3:** Transcripts annotated as paralogous genes with a known role in teleost development, where only one paralog was detected in the present study.

Two paralogs found in three-spined stickleback in Ensemble and/or Fish-it			One paralog found in three-spined stickleback in Ensemble and/or Fish-it		
Gene ID^b^	Chr.	References	Gene ID^b^	Chr.	References
st8sia3*	XIV (XIII)	^74^	ppar beta*	XII	^65^
ahr1*	Ι (XVI)	^75^	uts2a*	XII	^76^
dag1*	XVII (XII)	^77^	gli2*	XVI	^28^
marcks*	XVIII (XV)	^78^	mustn1b*	XVII	^79^
ncam1*	VII (I)	^80^	bmp2b*	XVIII	^81^
*stard13*	VII (I)	^82^	cart1*	XV	^83^
otx2*	XV (XVIII)	^84^	zic2a*	XVI	^21^
otx5*	I (VII)	^84^	mgrn1b*	XI	^21^
*tshr*	XVIII (XIV)	^85^	hoxc6α*	XII	^31^
nme2a*	scf48 (Un)	^86^	hoxc11α*	XII	^31^
*irf4*	VIII (III)	^87^	hoxc12α*	XII	^31^
prox1*	XVIII (VII)	^88^	hoxc13α*	XII	^31^
rab32*	XVIII (XV)	^21^			
pabpc1*	X (ΧΧ)	^21^			
ednrb1*	III (XVI)	^21^			
atp6v1e1*	IV (XIX)	^21^			
*kit*	VIII (ΙΧ)	^21^			
frem2*	I (VII)	^21^			
*myo7a*	I (VII)	^21^			
trpm1*	XIX (II)	^21^			
egfr*	III (scf 182)	^21^			
hoxb1b*	V (XI)	^31^			
hoxb3a*	XI (V)	^31^			
*hoxb6a*	XI (V)	^31^			

Transcripts that were differentially expressed between at least two stages are noted with an asterisk.

**Figure 6:**
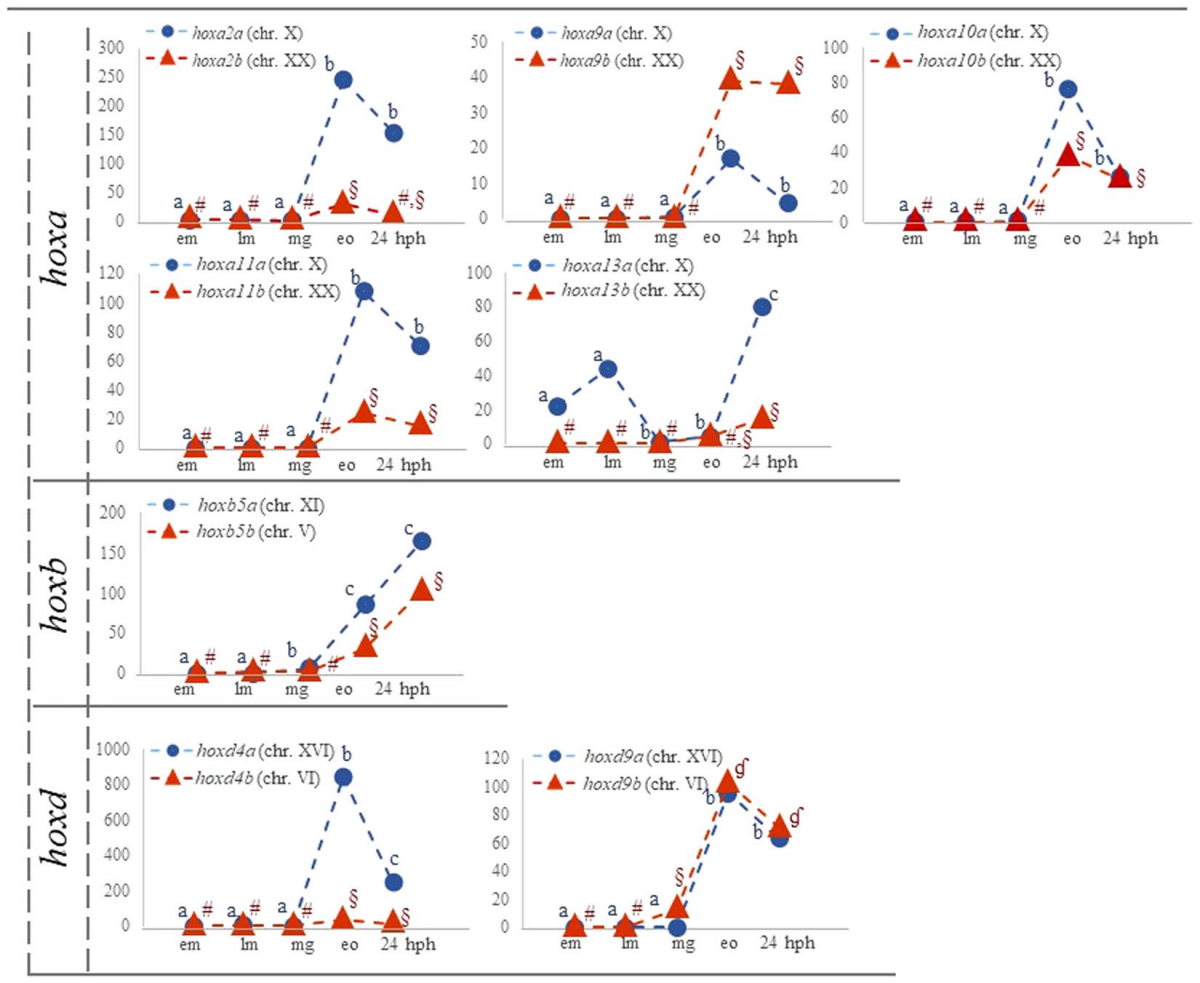
Expression pattern of *hoxa*, *hoxb* and *hoxd* paralogs during three-spined stickleback development. The y-axis shows normalized count values and the x-axis shows the five developmental stages studied. The expression pattern of the alpha paralog is illustrated with blue and of the beta paralog with orange line. The chromosome where each paralog was mapped is shown in parenthesis. Significant different expression values are marked with (**a**–**c**) in case of alpha paralog, and #, §, ʠ, ʡ in case of beta paralogs.

**Figure 7:**
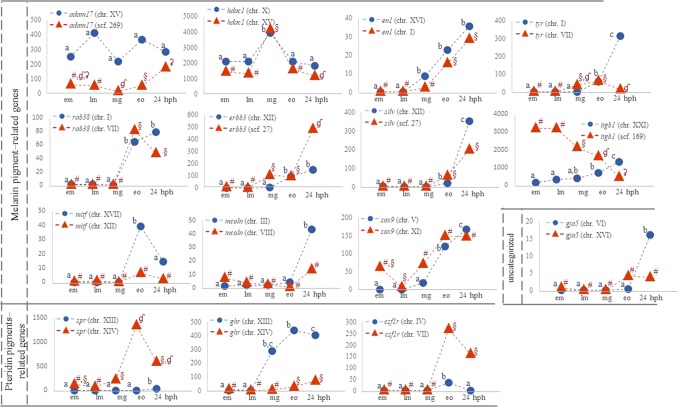
Expression pattern of genes implicated in animal pigmentation, that were found during three-spined stickleback’s development in more than one copy. The y-axis shows normalized count values and the x-axis shows the five developmental stages studied. The chromosome where each paralog was mapped is shown in parenthesis. Significant different expression values are marked with (**a**–**c**) in case of alpha paralog, and #, §, ʠ, ʡ in case of beta paralogs.

First, we focused on the presence of *hox* paralogous genes, which belong to a well-established group of duplicated genes with evolutionary and developmental interest^20^. *Hox* genes were searched among all annotated transcripts. In the present study, all genes of both *hoxa* clusters of three-spined stickleback were present. On the contrary, genes of *hox* clusters *b*, *c* and *d* (*hoxb1a*, *-2a*, *-3b*, -*6b*, *-7a*, -*8a*, *hoxc3*, *hoxd11a* and -*11b*) were not found (Supplementary Table S7). The expression patterns of duplicated *hox* genes are illustrated in Fig. 6. The majority of the studied *hox* genes were more highly expressed at early organogenesis/first appearance of somites stage and at 24 hph stage. During the earlier developmental stages (early and late morula) only *hoxa13a* were expressed, while the other members of the family were roughly present. Similarly, expression levels of another group of genes, those involved in body pigmentation, and of their paralogs when present, were also searched in the annotated dataset. The list of genes that participate in body pigmentation (Supplementary Table S8), as well as their categorization depending on the pigmentation pathway (melanin-, pteridine- and iridophore- related genes), was based on the study of Braasch *et al*.^21^. Figure 7 illustrates the expression pattern of pigmentation genes found to be duplicated in the present study. Eight of the genes studied had similar expression patterns with their paralog (*hdacI*, *en1*, *sox9*, *erbb3*, *silv*, *mcoln3*, *mitf*, and *csf1r)*. Notably, decreased expression of one paralog concurred with increased levels of the other paralog in the case of *ty*r, *rab38*, *gja5* and *itgb1* of the melanin-pigment related genes and *spr* of the pteridin-pigment related genes.

## Discussion

The present study investigated the expression patterns of five key developmental stages of the three-spined stickleback with a focus on paralogous genes. To primarily establish defined stage-specific “expression fingerprints”, the transcriptome patterns during early embryogenesis were explored. The generated transcriptome profiles were used to evaluate the sampling and sequencing procedures. First of all, the three biological replicates of each stage as illustrated in Fig. 1a were grouped together and separated from the other developmental stages validating effective staging and distinct transcriptomic profiles for each stage. Furthermore, a similar percentage of null counts and numbers of reads for each sample indicated comparable sequencing data between replicates of each stage and between stages (Supplementary Fig. S1).

To characterize the functionality of a tissue, it has been shown that a short list of the 10 most expressed transcripts is sufficient^22^. Abundance analysis in the present study revealed that the most abundant transcript in the first two stages studied (early and late morula) was the *cytochrome oxidase I*, most likely serving the metabolic needs for energy requirements through a period of intense divisions. The increased metabolic demands of the early and late morula were in agreement with the observation that, after fertilization, fish embryo oxygen consumption increases rapidly^23^. The increase in the number of cells during early and late morula, as the first embryonic task immediately after fertilization, is also shown by the over-representation of genes involved in molecular mechanisms related to the cell cycle, mitosis (e.g. regulation of G2/M transition of mitotic cell cycle) and chromatin separation (e.g. centromere complex assembly, attachment of spindle microtubules to kinetochore) (Supplementary Table S4). Similar results were shown in a zebrafish transcriptome profile study, during 1–16 and 512 cell stage^24^. In the third stage studied (the mid-gastrula/50% epiboly stage), the most expressed transcript was *efα*, which is related to the translational machinery^25^ (Fig. 1b). Similarly in the zebrafish at 50% of epiboly stage, *efα* was also among the 10 most expressed transcripts, serving the translational needs of the embryo^24^. In addition, GO terms unique in module 2 i.e., transcripts mainly upregulated during the mid-gastrula /50% epiboly, also signalled the next developmental leap: the initiation of organ development (Supplementary Table S4). Although biological processes that set the basis for organogenesis were already present in mid-gastrula/50% epiboly, from what is known, the major part of organogenetic processes is accomplished during the next developmental stage, the organogenesis^26^. Consistent with this, the transcripts mainly upregulated in early organogenesis/first appearance of somites stage (module 3, Fig. 4c) in the three-spined stickleback were mostly associated with GO terms relevant to organ development and morphogenesis (e.g. cardiac muscle, appendage, kidney vasculature, pectoral fin, neuron projection, blood vessel, rhombomere 4) (Supplementary Table S4). The last studied stage in the present work was revealed to be transcriptionally the most diverse one, as shown by the high degree of differentiation of this stage in the PCA analysis (Fig. 1a) as well as in the high total number of differentially expressed genes compared to all the other stages (Fig. 2). A major developmental step acquired at this stage is that movement is not a reflex but a conscious process. The detection of *actin* as the most abundant transcript in the three-spined stickleback’s 24 hph stage (Fig. 1b), appears to be related to the shift from the immobile embryo to the free-moving larvae since actin is a major component of skeletal muscle.

Having established the molecular bases in the form of differentially expressed transcripts among distinct developmental stages, paralogous genes either with different, similar or identical expression patterns among them were identified. Paralogs found in the present study and known to be involved in developmental processes are listed in Tables 2 and 3. Table 2 comprises the cases where both paralogs were found to be present and differentially expressed, while in Table 3 only those paralogs are listed where only one of the paralogs was detected. The occurrence of a single paralog may indicate that only one of the paralogs has a functional role during early development. On the other hand, twelve transcripts were found as a single copy in the present dataset as well as in any of the publicly available databases of the three-spined stickleback. As both paralogs were found in other fish species (Table 3), such as the zebrafish, it may be hypothesized that they have lost their counterpart in the three-spined stickleback. Zebrafish belongs to the Ostariophysi while the three-spined stickleback belongs to the Acanthopterygii. The two superorders were dated to have split approximately 217 ± 4 Ma ago^27^. In the group of Acanthopterygii eleven of the transcripts were found only in one copy (*mustn1b; uts2a; bmp2b*, *ppardb/pparbb*, *cart1*, *mgrn1b*, *zic2a*, *hoxc6a*, *hoxc11a*, *hoxc12a and hoxc13a*), while in the Ostariophysi its paralogs (*mustn1a; uts2b; bmp2a; pparda/pparba*, *cart1 second copy*, *mgrn1a*, *zic2b*, *hoxc6b*, *hoxc11b*, *hoxc12b and hoxc13b*) were detected. It has recently been shown that the Ostariophysi and the Acanthopterygii have a different paralogous genes retention rate, and seven of the twelve genes i.e., *bmp2*, *mgrn1*, *ppard/pparb*, *hoxc6*, *hoxc11*, *hoxc12 and hoxc13* belong to the lineage-specific paralogs (LSP)^7^. Thus, this may also explain the absence of *uts2b*, *cart1 second copy*, *zic2b and mustn1a* in the three-spined stickleback. With regard to *gli2*, its paralog has been identified in other teleosts belonging to the Acanthopterygii but to the best of our knowledge, not in the three-spined stickleback (Supplementary Fig. S3). *Gli2* is known to be a major mediator of the hedgehog signalling pathway in early development^28^. Studies in zebrafish have hypothesized that the paralog *gli2a* is superfluous in teleost fish^29^. However, with only one paralog retained i.e., *gli2a*, clearly the alpha paralog is indispensable in the three-spined stickleback.

Early development is a key life period of any organism including teleosts and the *hox* genes are among the well-studied duplicated genes crucial for proper development^30^. So far, in the three-spined stickleback, 48 *hox* genes have been identified^31^, located in seven clusters (*aa*, *ab*, *ba*, *bb*, *ca*, *da* and *db*)^32^. In the present study 39 *hox* genes were found, out of which 16 were paralogs with specific expression patterns as illustrated in Fig. 6. In general, both paralogs showed similar expression pattern with higher expression of the *hoxa* alpha paralogs (chromosome X) in four cases (*hoxa2a*, *hoxa10a*, *hoxa11a*, *hoxa13a*), while in one case the beta paralog (*hoxa9b*; chromosome XX) was only slightly more highly expressed. With the exception of *hoxa13a* (located on chromosome X), low or no expression of the *hox* genes under study was detected during the early stages. The majority of *hox* genes reached a peak at early organogenesis/first appearance of somites stage when the shaping of the body into distinct parts is initiated. This may be justified by the fact that the functional role of *hox* genes lies in the determination and specification of the body segments^33^.

Further investigations in the present study were focused on the differentially expressed genes involved in pigmentation, where for eight genes, (*mitf* (both paralogs), *csf1r* (both paralogs), *spr (*paralog chromosome XIV), *tyr* (paralog chromosome VII), *rab 38* (paralog chromosome VII) and *ghr* (paralog chromosome XIII) one distinct expression peak is seen at the early organogenesis/first appearance of somites (Fig. 7). Among them, three genes (*ghr*^21^, *csf1r*^34^, and *spr*^32^) are involved in the pteridin pigment synthesis, one of the two major synthetic pathways of pigments in the teleost. For each of the three paralog pairs, one paralog was found to be predominantly expressed. For example, the *ghr* paralog in chromosome XIII was already elevated at mid-gastrula/50% epiboly stage, when the neural crest is formed. In medaka, it has been shown that *ghra* (according to^21^ homologue to *ghr* paralog in chromosome XIII) binds more effectively somatolactin-α, a pituitary secreted hormone that is implicated in chromatophore development^35^.

Concerning the genes involved in the melanin-pigment synthesis, the second major synthetic pathway of pigments in the teleost, predominant expression of only one paralog has been detected for *mitf*^36^ (paralog on chromosome XVII) and *tyr* (paralog on chromosome I) with an expression peak at early organogenesis/first appearance of somites and at stage 24hph respectively (Fig. 7). This may be explained by the need to serve pigmentation processes as e.g. *tyr* is expressed in developing retina early in zebrafish embryogenesis and preceding melanin accumulation by a few hours^37^. Zebrafish, however, possesses only a single *tyr* gene located in chromosome 15 (the homologous group to the three-spined stickleback I)^21^.

Opposite expression patterns were found in two of the paralog pairs i.e., *gja5*, and *itgb1*. Concerning the complementary expression of *gja5* at the 24hph stage, it has been shown that the beta paralog is involved in an adult mutant zebrafish leading to a spotted (instead of striped) pattern. On the other hand, knockdown of the alpha paralog had no effect on the zebrafish skin pattern^38^. In the case of *itgb1*, also known as *cd29*, a particular pattern was observed. The two paralogs displayed a complementary expression throughout the developmental stages studied. *Itgb1* encodes for a cell surface receptor and is involved in the melanophore development^21^. Even though *itgb1* has been connected to melanocyte distribution and normal skin pigmentation in humans^39^, expression studies in teleost concerning their role in skin colour are absent. In mammals, it has been reported that *itgb1* plays a role in the junctions between germ and Sertoli cells during spermatogenesis^40^. The present finding that the one *itgb1* paralog is highly expressed at the early stages but decreases later on, while the other paralog increases, could provide novel significant insights worthy of further investigation and might pinpoint to a possible paralog sub-functionalization event.

## Conclusion

 In this paper, we demonstrated that distinct transcriptome profiles amongst developmental stages exist in the three-spined stickleback. The adjacent stages exhibited more similar expression patterns whereas the more distant stages revealed completely different profiles. We further identified paralogs differentially expressed during ontogenesis and demonstrated that paralogous genes, which are known to be involved in teleost development, also have varying expression patterns, with one of the paralogs being dominant and, in some cases, even the only one present. Untangling the specific paralog functions might not only significantly contribute to the understanding of teleost ontogenesis but might also shed light on paralogous genes’ evolution.

## Materials and Methods

The entire workflow followed in the present study is graphically shown in Supplementary Fig. S4.

### Ethical statement

Sampling was carried out at Cefas according to UK legislation. Up until the time point of the samples in our study, sticklebacks have yolk sacs and as such are not considered to be free feeding. Under UK legislation, fish embryos become protected under the Animals (Scientific Procedures) Act from the free feeding stage onwards.

### Sampling

Three-spined stickleback fertilised eggs and embryos were collected in triplicate at different time periods from the well-established laboratory colony maintained at the Centre for Environment, Fisheries and Aquaculture Science, at Weymouth, UK. Eggs were collected from a single gravid female via mild abdominal pressure and were fertilised by the sperm of a single male within 1-minute post collection. The fertilised eggs were examined for quality under a microscope and placed in 1 L glass flasks containing dechlorinated freshwater, under aeration (air stone) at 18 °C, 30 minutes post fertilisation. In total, five developmental stages were collected comprising of the: i) early morula, ii) late morula, iii) mid-gastrula/50% epiboly, iv) early organogenesis/first appearance of somites and v) 24 hours post-hatching. The staging observation was performed according to Swarup^41^. After designation of the exact developmental stage, all samples were immediately frozen in liquid nitrogen and stored at −80 °C until they were dispatched in dry ice to the Institute of Marine Biology, Biotechnology and Aquaculture at the Hellenic Centre for Marine Research in Crete, Greece.

### Total RNA extraction

Total RNA of all samples was extracted using Nucleospin miRNA kits (Macherey-Nagel, Düren, Germany) according to the manufacturer’s instructions. Briefly, pools of eggs or embryos were disrupted with a mortar and pestle in liquid nitrogen and homogenized in lysis buffer by passing the lysate through a 23-gauge (0.64 mm) needle five times. The quantity of extracted RNA was estimated using a NanoDrop ND-1000 spectrophotometer (NanoDrop Technologies, Wilmington, DE). The RNA quality was further evaluated by agarose (1%) gel electrophoresis, as well as by capillary electrophoresis using the RNA Pico Bioanalysis chip (Agilent 2100 Bioanalyzer, Agilent Technologies, Santa Clara, CA 95051, USA). Samples with an RNA integrity number value between 8.9 and 9.9 were used for library construction.

### Library preparation and sequencing

Fifteen mRNA libraries (three libraries per developmental stage) were prepared using Truseq stranded mRNA library preparation kit (Illumina, 5200 Illumina Way, San Diego, CA 92122 USA). The magnetic-bead-assisted mRNA purification was followed by mRNA fragmentation. First and second strand synthesis produced cDNA, which was ligated with different adapters, one for each library. Libraries were then amplified by PCR and validated by capillary electrophoresis using a High Sensitivity DNA chip (Bioanalyzer, Agilent Technologies). The quantification of each library was performed by qPCR, using the Kappa Library Quantification kit (Kappa Biosystems, Wilmington, MA 01887, USA). Libraries were paired-end sequenced (150 bp) over two lanes using Illumina HiSeq vs. 2500 at the Norwegian Sequencing Centre, Oslo, Norway.

### Read quality, mapping to genome and transcriptome assembly

Sequencing reads generated for each library were initially checked for their quality using FastQC (v0.11.5)^42^. For adaptor sequences as well as low-quality nucleotide reads removal, Trimmomatic (v. 0.32)^43^ was used. Cleaned sequencing reads were mapped to the three-spined stickleback genome (Gasterosteus_aculeatus.BROADS1.dna.toplevel.fa, http://ftp.ensembl.org/pub/release-76/fasta/gasterosteus_aculeatus/dna/) applying CRAC (v. 2.5.0)^44^, a software for mapping RNA sequencing reads to the genome with high precision in splice junctions. Genome-guided assembly was performed using Stringtie (v. 1.2.2) assembler^45^ in two steps: the first step assembled the mapped reads of each RNA-Seq sample separately. The resulting assemblies were then merged, at the second step, into one transcriptome containing unique sequences.

### Annotation

Annotation of the assembled transcriptome was performed by i) mapping reads to the genome of the three-spined stickleback with default parameters, ii) submission to blastx search against the nr database of NCBI with E-value < 1 10^−8^ and iii) using Blast2GO platform (v 4.1.9) with default parameters^46^. Blast2GO platform was also used for gene ontology (GO) terms assignment.

### Differential expression

Trimmed reads of each stage were mapped to the constructed transcriptome with the RSEM estimation method (with align and estimate abundance.pl script of Trinity, r20140717); (parameters used: “RF” library type, “fq” sequence type, bowtie as aligning method)^47^. The generated count matrix served as an input file for differential expression analysis, following the DESeq2 pipeline^48^ integrated into SARTools^49^. After pair-wise comparison, transcripts with padj <0.005 and log2fold change ≥|2| between two stages were considered as differentially expressed.

### Data evaluation

Principal component analysis (PCA) was used to evaluate the quality of the whole data matrix using plot3D function in R. Furthermore, sample clustering was performed with WGCNA (Weighted Correlation Network Analysis) to detect outliers and group all differentially expressed transcripts according to their expression pattern, using the “block-wise network construction and module detection” R script which is appropriate for large datasets^50^. Heat maps were constructed with heatmap.2 function in R, using normalized count data of differentially expressed transcripts.

### Enrichment analysis

Two-tailed enrichment analysis with default parameters (filter value: 0.05 filter mode: FDR, two-tailed) was performed applying Blast2GO (v 4.1.9) with padj ≤0.05 filter^46^. All the annotated transcripts were used as a reference dataset and modules 1, 2, 3 and 4 that counted the majority of the transcripts (Fig. 3) were used as test set.

### Paralog identification

To detect paralogous genes, extra gene copies were examined in all differentially expressed transcripts between different developmental stages of the three-spined stickleback’s developmental stages. In the present study, two genes were considered as putative paralogs if they combined the following: i) they shared identical or similar (-like) annotation terms and ii) they mapped in different chromosomes or linkage groups. In addition, candidate paralogs were searched among three-spined stickleback paralogs in the Ensembl Compara database.

## Supplementary information

Supplementary Tables and Figures

Table S3

Table S4

Table S5

Table S6

Table S7

Table S8

## Data Availability

All reads resulting from Illumina sequencing were submitted to the public database of SRA (Sequence Read Archive) of NCBI under the PRJNA395155 Bioproject, comprising 15 biosamples (one biosample for each library) coded from SAMN07374938 (early morula, replicate 1) to SAMN7374952 (24 hph, replicate 3). This Transcriptome Shotgun Assembly project has been deposited at DDBJ/EMBL/GenBank under the accession GHCM00000000. The version described in this paper is the first version, GHCM01000000.
