# Supplementary material for: Unravelling paralogous gene expression dynamics during three-spined stickleback embryogenesis
Source: Sci Rep. 2019 Mar 6;9:3752. doi: 10.1038/s41598-019-40127-2 (PMC6403355; doi:10.1038/s41598-019-40127-2)
Supplement: Supplementary file 1 — Supplementary Tables and Figures [file 41598_2019_40127_MOESM1_ESM.pdf]

Unravelling paralogous gene expression dynamics during three-spined stickleback embryogenesis

***Kaitetzidou Elisavet*[1,2], *Katsiadaki Ioanna*[3], *Lagnel Jacques*[2,4], *Antonopoulou Efthimia*[1], *Sarropoulou Elena*[2*]**

[1]Department of Zoology, School of Biology, Faculty of Sciences, Aristotle University of Thessaloniki, Greece.
[2]Institute for Marine Biology, Biotechnology and Aquaculture, Hellenic Centre for Marine Research, Greece.
[3]Centre for Environment, Fisheries and Aquaculture Science (Cefas), Weymouth, UK.
[4]Institut National de la Recherche Agronomique (INRA), Génétique et Amélioration des Fruits et Légumes (GALF), Allée des Chênes, Montfavet Cedex, France.

*Correspondence to sarris@hcmr.gr

Supplementary Table S1: Number of reads of each sample, as well as total number of reads, produced after sequencing (paired-end raw reads), and after quality trimming (trimmed reads), as well as the percentage of reads which passed the quality trimming (% percentage).

	paired-end raw reads	trimmed reads	% percentage
early morula 1	13,006,307	10,805,525	83.08%
early morula 2	19,420,988	15,373,722	79.16%
early morula 3	23,380,632	19,584,634	83.76%
late morula 1	14,338,040	11,814,467	82.40%
late morula 2	15,273,077	12,570,807	82.31%
late morula 3	23,902,779	20,094,200	84.07%
mid gastrula/ 50% epiboly 1	14,785,565	12,742,859	86.18%
mid gastrula/ 50% epiboly 2	16,328,561	14,102,138	86.36%
mid gastrula/ 50% epiboly 3	20,076,756	17,224,342	85.79%
early organogenesis/ first appearance of somites 1	19,383,635	16,721,611	86.27%
early organogenesis/ first appearance of somites 2	20,357,066	17,673,379	86.82%
early organogenesis/ first appearance of somites 3	19,375,722	17,227,912	88.91%
24 hph 1	18,535,463	15,549,240	83.89%
24 hph 2	18,068,980	15,011,261	83.08%
24 hph 3	20,285,215	16,694,369	82.30%
**total**	**276,518,786**	**233,190,466**	**84.33%**

Supplementary Table S2: Numeric information of the resulted assembled transcriptome.

Total number of sequences	101,296
Average length (bp)	1,7904.98
Minimum length (bp)	200
Maximum length (bp)	78,398
N50	2,608

Supplementary Figure S1:

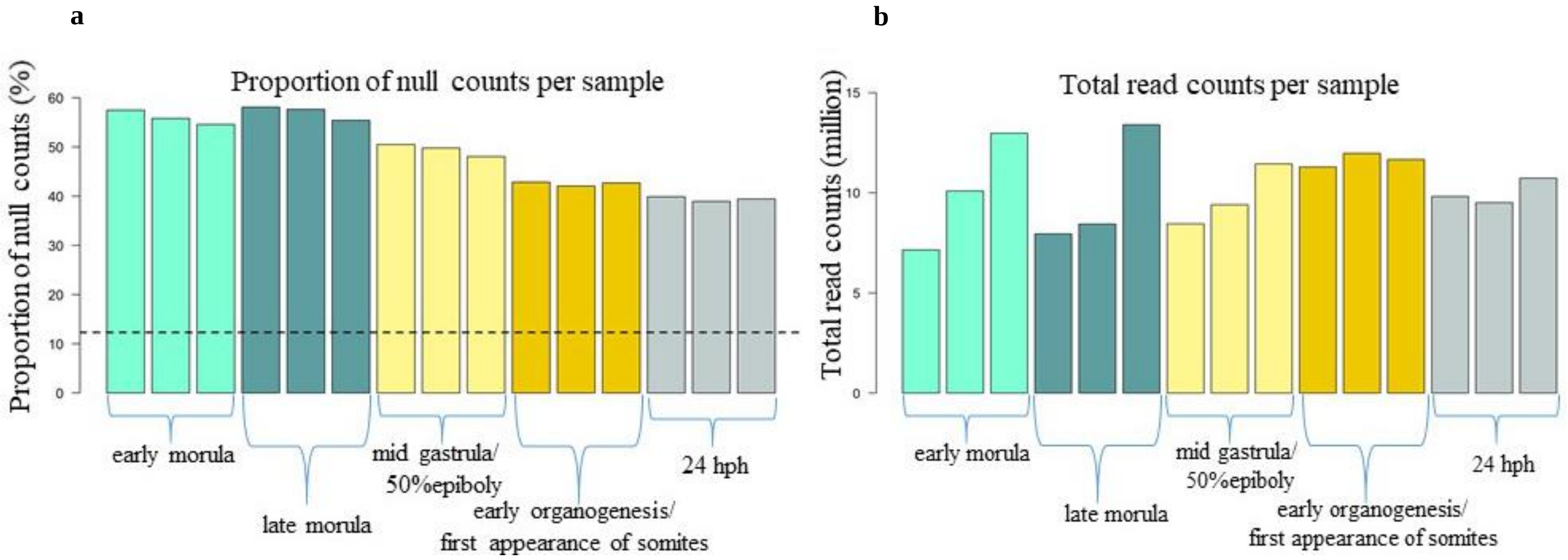

Figure S1: Information regarding null counts as well as total number of reads for each sample: a) The proportion of null counts in each sample showed that replicates of each stage, shared similar numbers. Also, as embryogenesis proceeded the number of null counts was lowering. b) Total read counts of each sample was also similar between replicates.

Supplementary Figure S2:

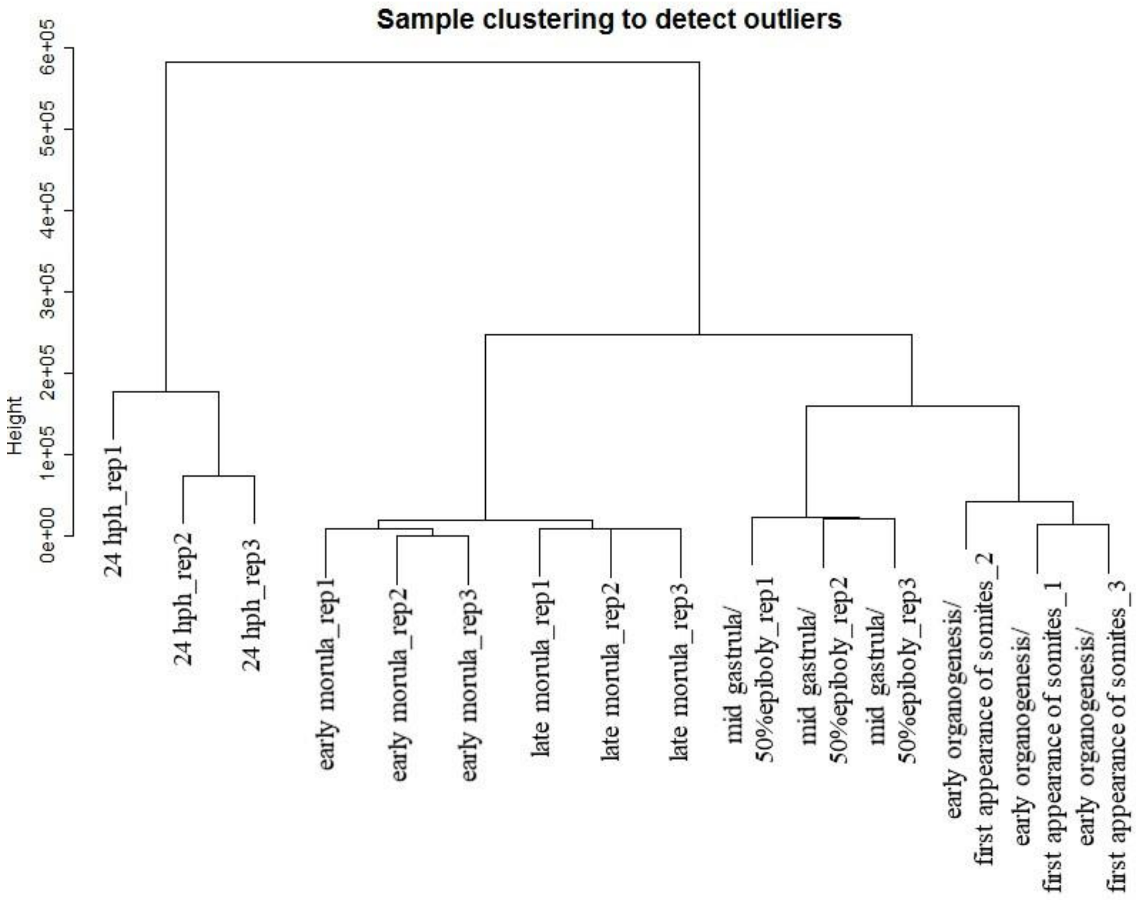

Supplementary Figure S2: Dendrogram of developmental stages based on the expression of differentially expressed transcripts: Dendrogram of developmental stages of stickleback after hierarchical clustering using differentially expressed transcripts (padj<0.005 and log2 fold change ≥ |2|). Replicates of each stage were clustered together. Moreover, developmentally closer stages shared the same branch of the dendrogram.

Supplementary Figure S3:

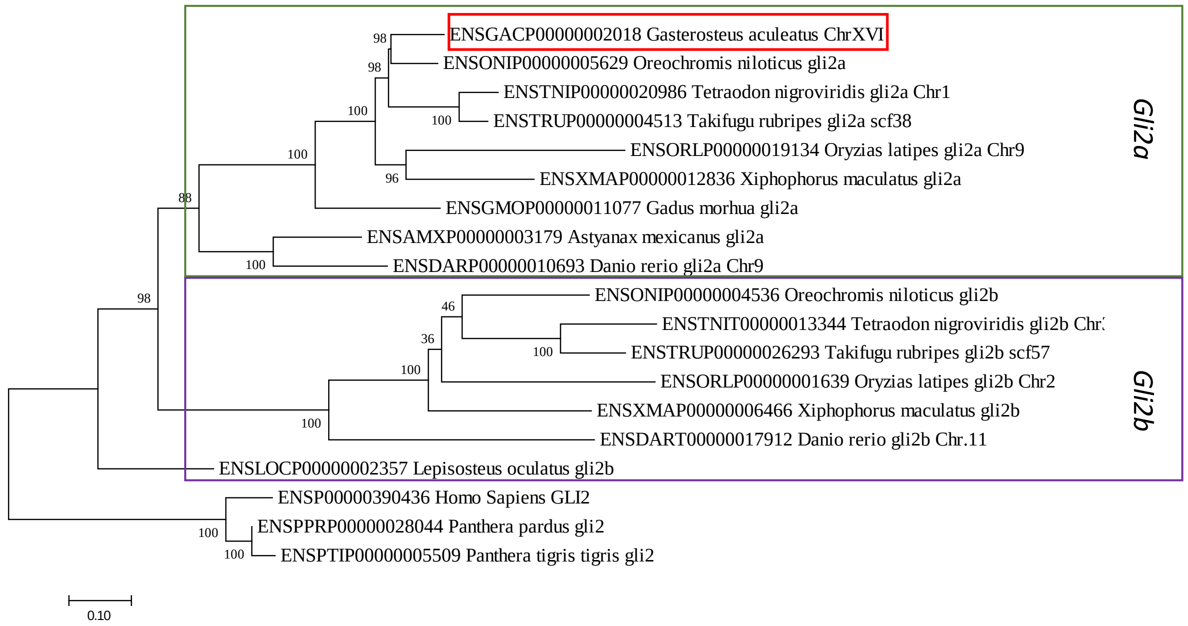

Supplementary Figure S3: Molecular phylogenetic analysis of *gli2* gene(s): Nine sequences annotated as *gli2a* and 7 sequences annotated as *gli2b* of 9 teleost species and spotted gar (*Lepisosteus aculeatus*), as well as 3 *Gli2* genes of mammals were used.

Supplementary Figure S4:

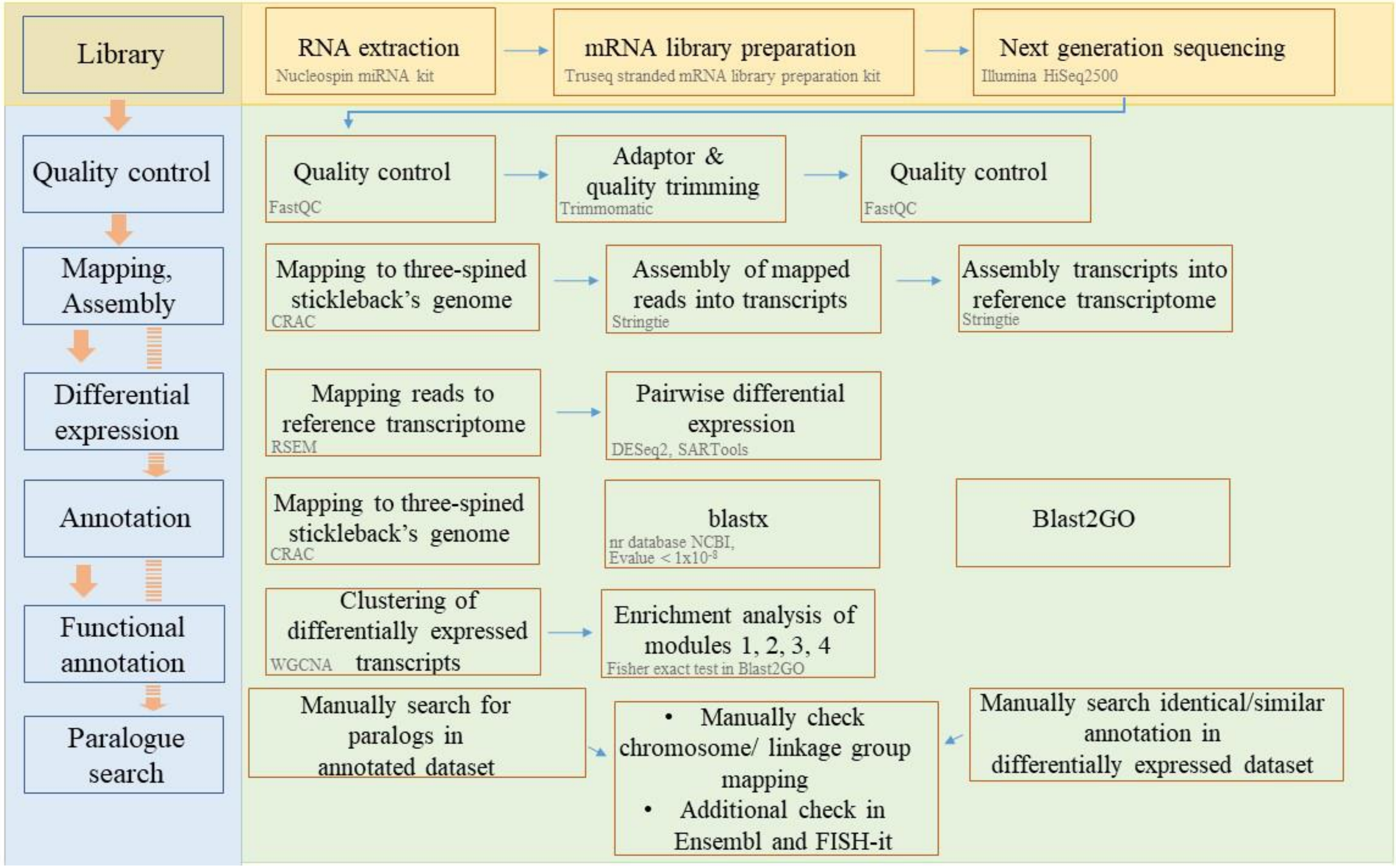

Supplementary Figure S4: The workflow that was followed through this study: from library preparation to differential expression, annotation analysis and paralog search.
